# Use of capnography for prediction of obstruction severity in non-intubated COPD and asthma patients

**DOI:** 10.1186/s12931-021-01747-3

**Published:** 2021-05-21

**Authors:** Barak Pertzov, Michal Ronen, Dror Rosengarten, Dorit Shitenberg, Moshe Heching, Yael Shostak, Mordechai R. Kramer

**Affiliations:** 1 The Pulmonary Division, Pulmonary Institute, Rabin Medical Center, Beilinson Campus, 49100 Petach Tikva, Israel; 2 Sackler Faculty of Medicine, Tel Aviv University, Tel Aviv, Israel; 3 Medtronic, Patient Monitoring, Jerusalem, Israel

**Keywords:** Asthma, COPD, Airway obstruction, FEV1, Capnography, Model, Neural network

## Abstract

**Background:**

Capnography waveform contains essential information regarding physiological characteristics of the airway and thus indicative of the level of airway obstruction. Our aim was to develop a capnography-based, point-of-care tool that can estimate the level of obstruction in patients with asthma and COPD.

**Methods:**

Two prospective observational studies conducted between September 2016 and May 2018 at Rabin Medical Center, Israel, included healthy, asthma and COPD patient groups. Each patient underwent spirometry test and continuous capnography, as part of, either methacholine challenge test for asthma diagnosis or bronchodilator reversibility test for asthma and COPD routine evaluation. Continuous capnography signal, divided into single breaths waveforms, were analyzed to identify waveform features, to create a predictive model for FEV1 using an artificial neural network. The gold standard for comparison was FEV1 measured with spirometry.

**Measurements and main results:**

Overall 160 patients analyzed. Model prediction included 32/88 waveform features and three demographic features (age, gender and height). The model showed excellent correlation with FEV1 (R=0.84), R^2^ achieved was 0.7 with mean square error of 0.13.

**Conclusion:**

In this study we have developed a model to evaluate FEV1 in asthma and COPD patients. Using this model, as a point-of-care tool, we can evaluate the airway obstruction level without reliance on patient cooperation. Moreover, continuous FEV1 monitoring can identify disease fluctuations, response to treatment and guide therapy.

**Trial registration:**

clinical trials.gov, NCT02805114. Registered 17 June 2016, https://clinicaltrials.gov/ct2/show/NCT02805114

## Introduction

Lung function measurement with a spirometer is considered the gold standard for assessment of asthma and chronic obstructive pulmonary disease (COPD) [1, 2]. Two important values measured by the spirometer, the value of Forced Expiratory Volume in 1s (FEV1) and the ratio of FEV1 to forced vital capacity (FVC) are used to assess the severity of airway obstruction. The measured FEV1 and FVC are normalized by the predicted FEV1 and FVC values, which depends on the patient's age, height, gender and ethnicity, and hence referred to as percent predicted FEV1 (%FEV1) and FVC [3, 4]. Spirometry can also be used to diagnose airway hyperresponsiveness (AHR); the methacholine challenge test (MCT) evaluates AHR by measuring FEV1 before and after the introduction of an increasing concentration of methacholine, an airway provoking agent, which in cases of AHR will cause a decrease in FEV1 of more than 20% [5]. However, spirometry requires cumbersome equipment, patient cooperation and technician experience [4], therefore, methods to evaluate lung function that are not reliant on patient cooperation and have reduced test retest variability are constantly being sought.

**Fig. 1:**
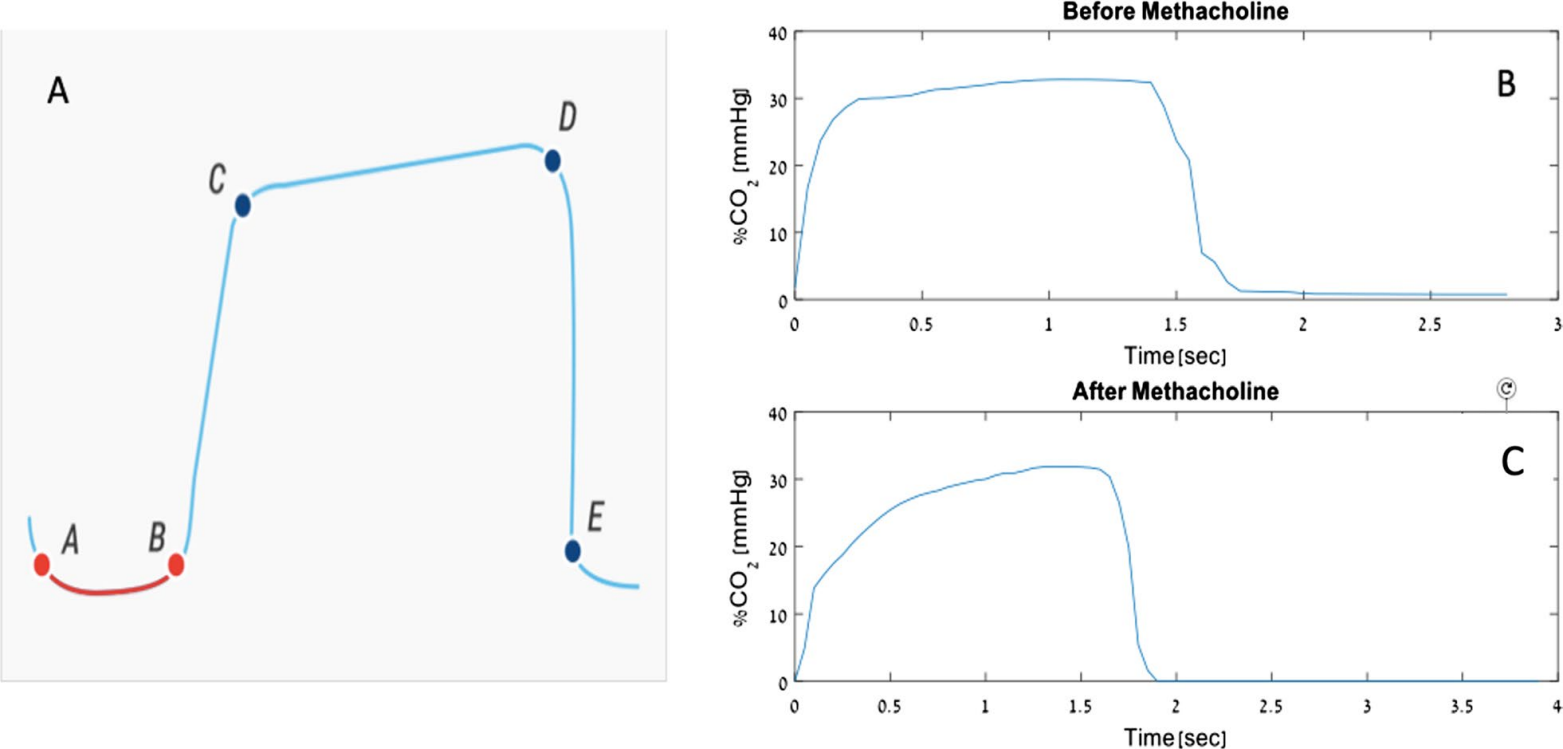
**A** Phases of CO_2_ Waveform. Capnography waveform readings during a positive methacholine challenge test. **B** Normal waveform without airway obstruction. **C** Capnography waveform after FEV1 decrease

Capnography is a waveform display of CO2 concentration in a gas mixture. It is often measured in intubated patients to confirm tracheal tube position and also to evaluate patient ventilation [6,8]. Capnography can also be measured in non-intubated patients using side-stream capnography; while the patient is breathing into a cannula, exhaled air is sampled and transferred through a long tube for processing. Graphic display of CO_2_ concentration over time depicts a distinct waveform, with 3 phases (Fig.1). Phase I (points A to B) reflects dead space ventilation in non-respiratory bronchi, which is normally devoid of carbon dioxide. Phase II (points B to C) reflects the arrival of gas from the respiratory bronchioles and alveoli. Gas diffusion of oxygen and CO_2_ occur in these parts of the respiratory system; hence CO_2_ concentration rises in the waveform. Phase III (points C to D) reflects the alveolar plateau, arrival of CO_2_-rich gas from the alveoli. Point D represents the end tidal CO_2_ (EtCO_2_), marks the end of expiration and the beginning of the inspiratory downstroke [9,11]. While capnography is currently used to monitor respiration rate and EtCO_2_, the CO_2_ waveform also contains essential information regarding physiological characteristics of the respiratory system. Several studies that investigated different utilization of the capnography waveform have shown a correlation between the waveform of the capnography curve and ventilation/perfusion mismatch, airway diameter and level of airway obstruction [12,16] (Fig.1). The aim of this study was to develop a capnography-based prediction model, to be used as a point-of-care tool that can evaluate the airway obstruction level in asthma and COPD patients.

## Methods

### Design

We conducted two observational prospective studies from September 2016 until May 2018 in Rabin Medical Center, Israel. The first cohort included healthy or asthmatic patients undergoing MCT for AHR evaluation. The second cohort included asthma and COPD patients with various levels of obstruction severity attending a routine ambulatory clinic. Each patient underwent a bronchodilator reversibility testing that is routinely performed for ambulatory asthma and COPD patients at the clinic evaluation. Both studies included adult patients who signed an informed consent form. Exclusion criteria were pregnancy, oxygen requirement of more than 5 L/min, lobectomy within the last year or patients who could not perform spirometry or capnography testing. The study was approved by the institutional review board (RMC-16-0412) clinical trial registryNCT02805114.

### Study protocol

#### Methacholine challenge test

The first stage included baseline spirometry and capnography measurements. After each stage of methacholine treatment [5] two minutes of capnography recordings were performed followed by spirometry. Patients that showed an obstructive response to the methacholine, defined as FEV1 values of 80% from baseline, were treated with a bronchodilator and the test was stopped.

#### Ambulatory clinic spirometry with bronchodilator reversibility

Included patients had a diagnosis of asthma or COPD, given by the treating pulmonologist and had a FEV1 of less than 80%. First, baseline spirometry and capnography were recorded, followed by treatment with a single dose of a short acting bronchodilator. After bronchodilator treatment, the patient was evaluated with capnography, followed by spirometry at 2, 4, 9, 12 and 15min.

### Data collection and processing

**Fig. 2:**
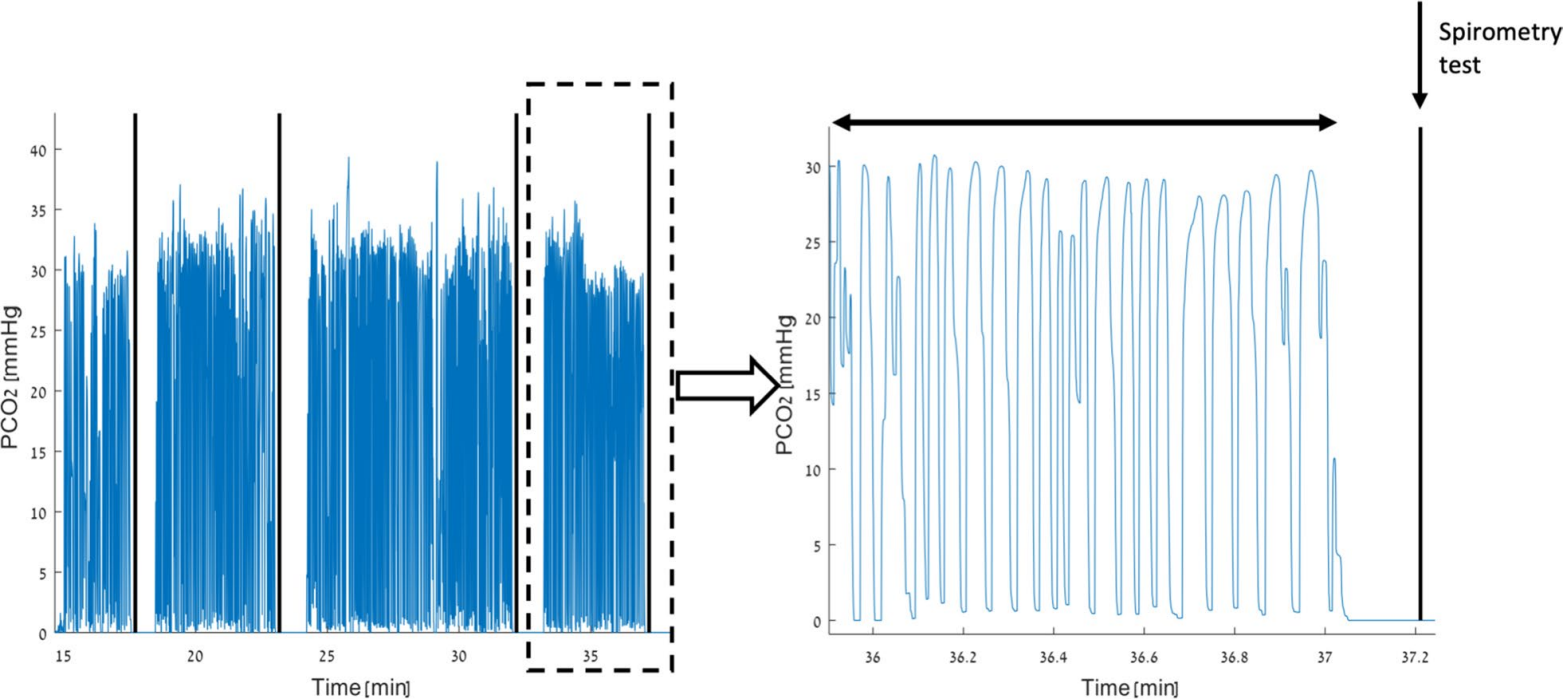
Data collection: For each segment of breath signals, the FEV1 at the end of the segment was considered as the gold standard reference value for obstruction level

Capnography data was collected with the Smart CapnoLine nasal canola connected to Capnostream 20p device. The equipment was supplied by Medtronic LTD (Dublin, Ireland) specifically for this study. Spirometry test were performed by a certified technician using a spirometry device at Rabin Medical Center. For data collection the capnography waveforms were continuously recorded. For data processing the continuous recording of multiple breaths were segmented into single breath signals. For each segment of breath signals, the FEV1 at the end of the segment was considered as the gold standard reference value for obstruction level, under the assumption that the obstruction level did not significantly vary between capnography measurement time window and the following spirometry time window (Fig.2). To improve classification accuracy, 7000 breaths signals were manually examined and the shape of the waveform was classified according to 8 features, as valid or non-valid. The data acquired manually was then used to create an artificial neural network (described below) classifier to exclude non-valid breaths in the entire data set. Breath segments were also evaluated; since all breaths in a segment were considered to have the same obstruction level, the waveform features of breaths in a segment should also be stable. Therefore, segments with a high number of breaths with extreme values or slops were removed. All data processing and analysis was conducted using MATLAB (R2016b, Mathworks ). To evaluate the model performance in patients treated with supplemental oxygen paired T test was used.

### Prediction model

#### Artificial neural network

An artificial neural network (ANN) is a general mathematical computing method that models the operations of biological neural systems. ANN is used for medical research in multiple disciplines, it is very useful for image recognition, decision making and model generation [17,20]. The basic unit in the network is called a neuron (or node) and it consists of two parts: the net function, which determines how the inputs are combined inside the neuron, and a non-linear mathematical function, called activation function. The network, comprised of many neurons, is organized in an interconnection structure, consists of input layer that includes the features or variables assumed to be the predicting factors, one or more hidden layers that manipulate the values according to the nodes functions and the output layer that gives a value that predicts the actual value given by the gold standard [21, 22]. The network learning is an iterative process that comprises of forward and back propagation; each forward propagation computes an output which is then compared to the actual value, the difference between the predicted and actual values is called the error function. Each back-propagation updates the weight of the nodes in a way that minimize the error function. After several cycles the ANN readjusts to compute the best predictive value. If the network architecture is too complex, e.g., has too many hidden units, or too many input features, then it may fit the noise and outliers, leading to overfitting. Such models perform well on the training set but poorly on new input [23]. To avoid overfitting, model parameters must be chosen correctly, with appropriate regularization technique.

### Model generation

For each breath signal, 88 features representing different characteristics of the waveform were extracted, based on the assumption that they may be responsive to the level of obstruction. Features of a specific breath waveform were extracted from either single breath features or multiple breaths features, from up to 10 consecutive breaths.

In addition to waveform features, age, gender and height were also attributed to each breath signal, as features.

ANN was trained, using a backpropagation algorithm, with training set features, to fit each breath to its %FEV1 value, representing the level of obstruction. Seventy percent of the data was randomly selected as the training set, leaving the remaining 30% for validation. Multiple combinations of parameters were formed with different values for each parameter. The network was trained according to each combination, using the back-propagation algorithm, to predict %FEV1. The process repeated itself several times, using Monte-Carlo cross validation process [24]. Each time different training and validation sets were randomly chosen. Each design, in each iteration was evaluated by the Mean square error (MSE) of the test set, between the model score and the %FEV1. MSE was averaged for each design over all iterations, and the best architecture was chosen as the one that achieved minimal MSE.

## Results

**Fig. 3:**
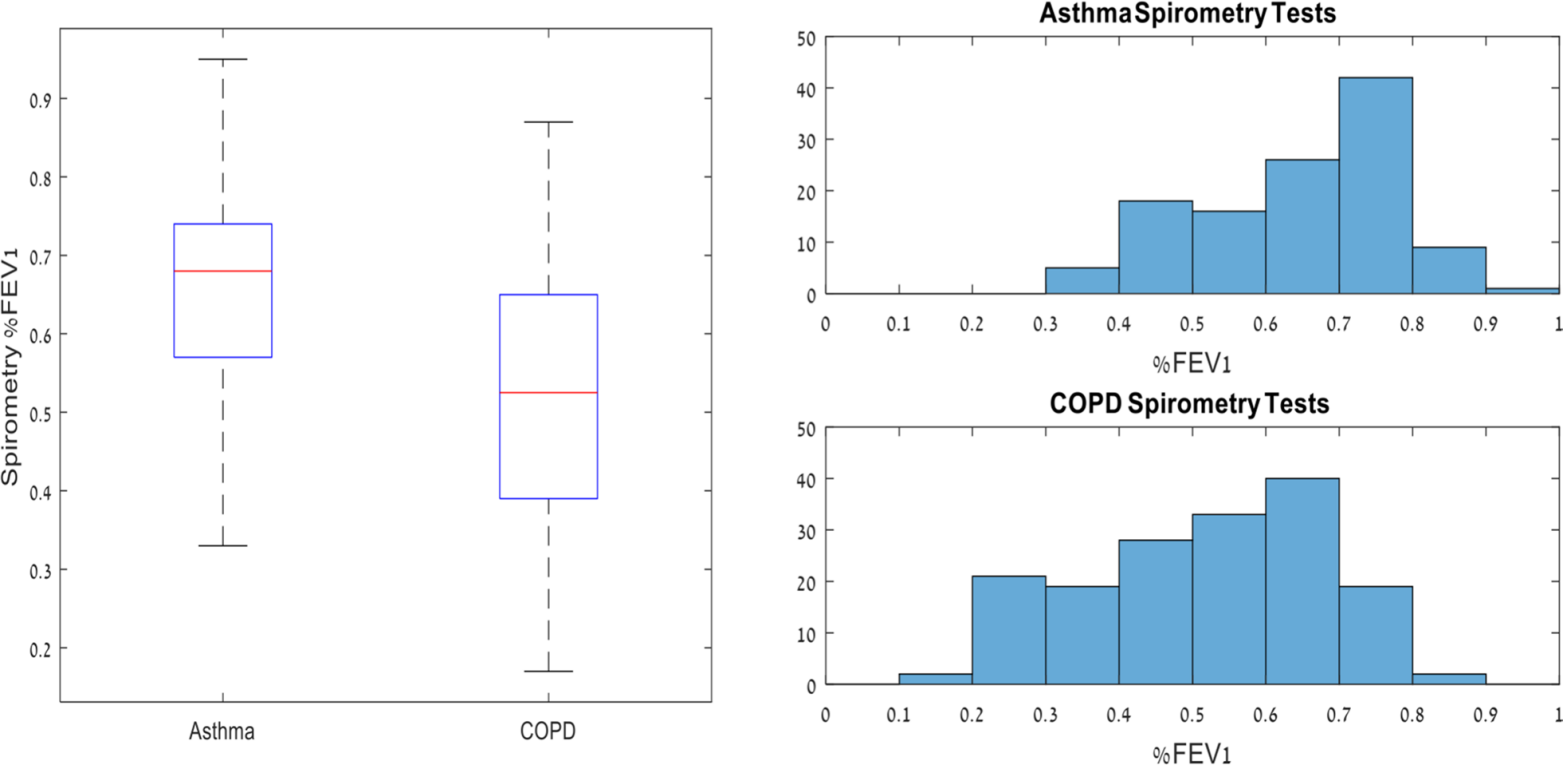
Spirometry results for included patients with asthma and COPD

**Fig. 4:**
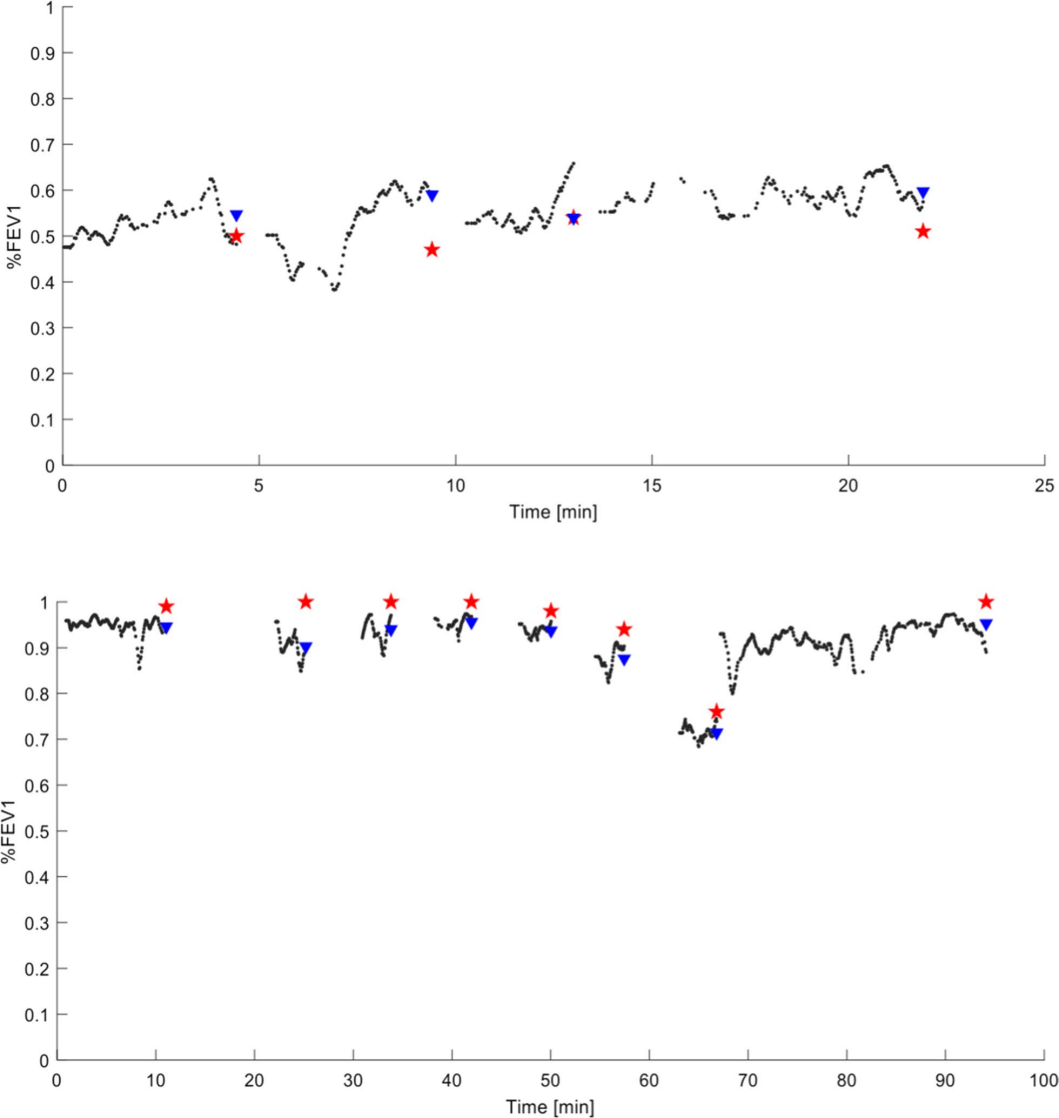
Model prediction results in two individual patients. blackmodel results per breath, Red star5min average of model resultsblue triangle dotspirometry value at the end of the segment

**Fig. 5:**
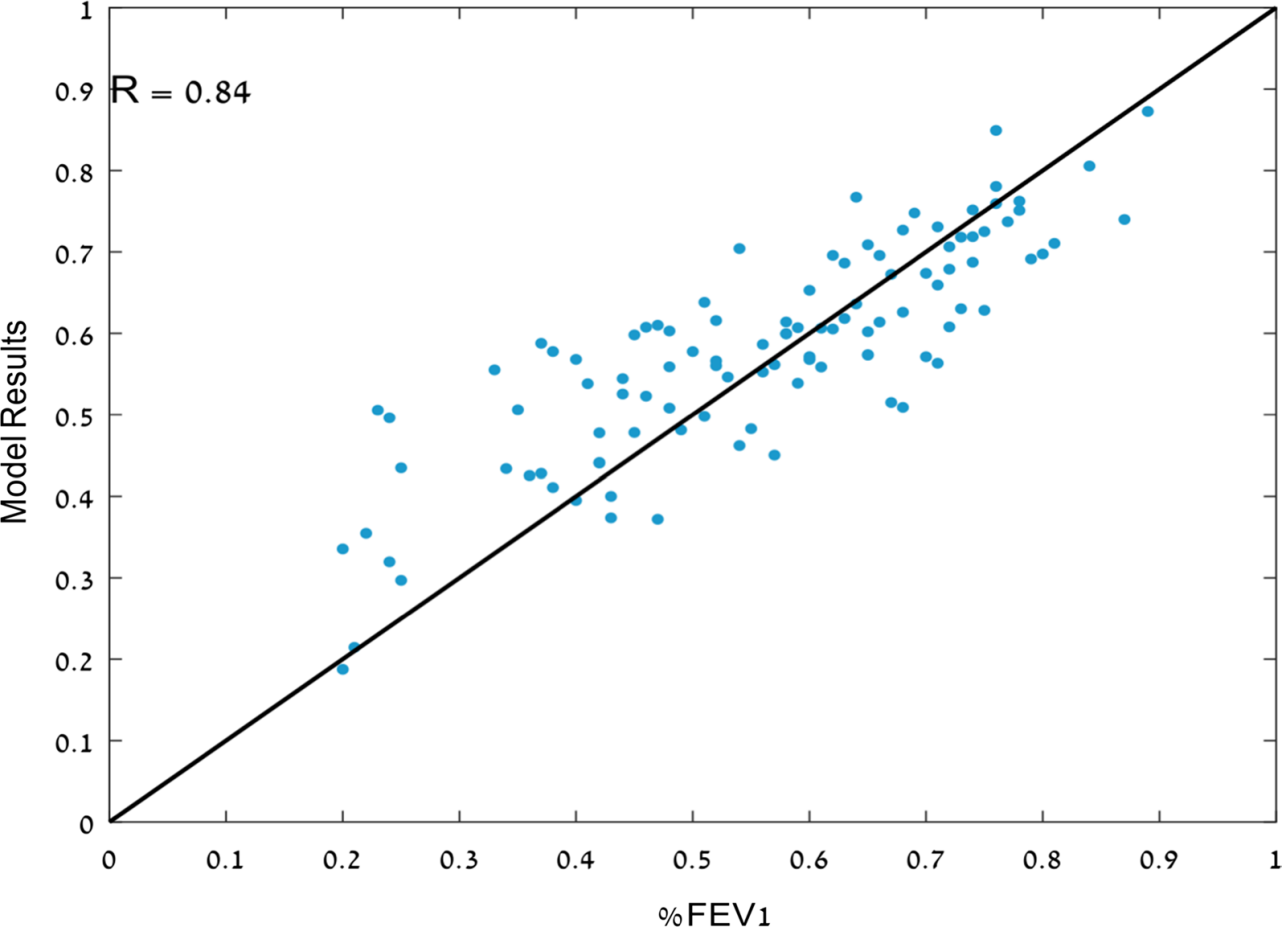
Correlation between FEV1 and Capnography Waveform Model

**Fig. 6:**
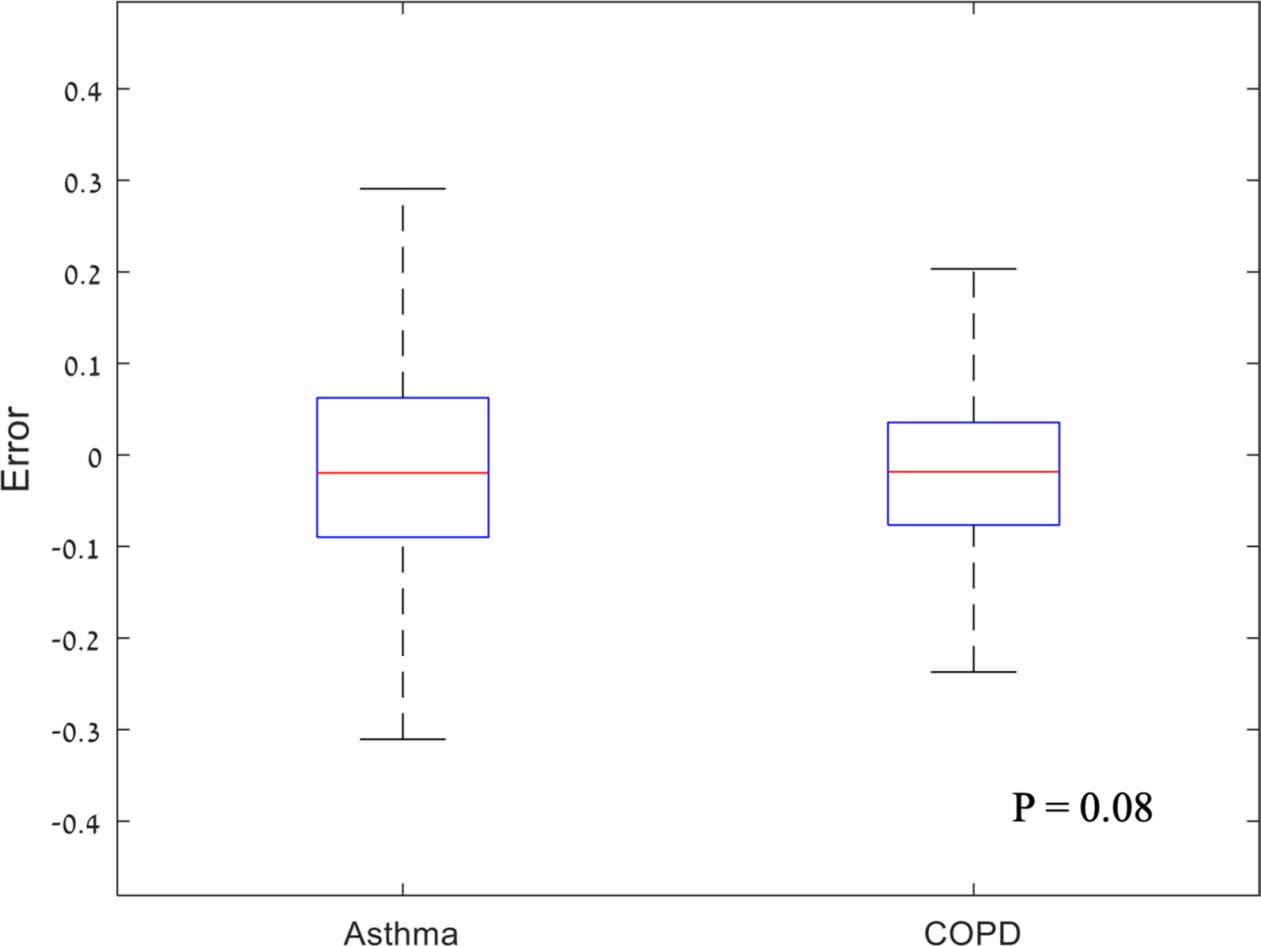
Error according to underlying disease

Overall, 160 patients were included in the analysis. In the MCT study, 60 patients were included, mean age was 47 (range 2074), 29 patients (48%) were male, 29 patients were diagnosed with AHR after the MCT. The bronchodilator reversibility study included 100 patients, mean age was 45(range 2086), 59 patients were male, 38 patients had asthma, 51 COPD and 11 patients had asthma and COPD overlap (Table 1). Disease severity was variable, the range of %FEV1 was 3090 and 1090 percent predicted for asthma and COPD, respectively (Fig.3). Of the 88 possible features for model prediction, 32 waveform features and three demographic features (age, gender and height) were included (Table 2). The model was generated with 1 hidden layer, 23 nodes and sigmoid activation function. Average scores from breaths during 5min prior to spirometry were calculated (Fig.4). The model showed excellent correlation with %FEV1 (R=0.84), the R^2^ achieved was 0.7 with MSE of 0.13 (Fig.5). Difference in the error distribution between asthma and COPD was evaluated and found non-significant (p=0.08) (Fig.6). The model was consistent across all levels of obstruction severity: with MSE of 0.06, 0.12, 0.11 and 0.15 for very severe (FEV1<30%), severe (FEV1 between 30 and 50%), moderate (FEV1 between 50 and 80) and mild (FEV1>80%) obstruction, respectively.

**Table 1:** Demographic and clinical characteristics

Study	N	Age (Mean [range])	Male gender (%)	Diagnosis			
				Healthy	Asthma	COPD	Astma+COPD
MCT	60	47 [2074]	29 (48)	31	29		
BRS	100	45 [2086]	59 (59)	0	38	51	11

*MCT* methacholine challenge test, *BRS* bronchodilator reversibility test; COPD: chronic obstructive pulmonary disease

**Table 2:** Model features

Feature	Description
1	Age
2	Gender
3	Height
4	Curvature of signal from start to D
5	Hjorth mobility
6	Ratio of areas from middle of signal to end over start to middle
7	Time spent in EtCO_2_ (with error of 0.01)
8	First index to reach EtCO_2_ (with error of 0.1) normalized by D
9	'Slope for C to D
10	Exponential FitMSE
11	Angle of the downward slope
12	Distance of point C from (0,1)
13	Linear regression from C to Da
14	Ratio between time signal rises and time signal descends
15	Distance from point C to the line connecting (0,0) to point D
16	Normalized signal at 0.5*D
17	First index to reach EtCO_2_ (with error of 0.1)
18	d2d1
19	Amount of times the signal rises
20	RW score1
21	Linear regression from 1 to DR2
22	Ratio between time until D/time from D to end (upstroke/downstroke)
23	Angle at C
24	Normalized signal at 0.45*D
25	Signal at the point where line from (0,1) to (1,0) crosses the signal
26	Ratio between signal rises/signal descends (from C to end)
27	Normalized signal at 0.35*D
28	Normalized signal at 0.4*D
29	Normalized signal at 0.55*D
30	Normalized signal at 0.3*D
31	Linear regression from C to Db
32	Like previous normalized by D
33	Normalized signal at D/2
34	Normalized signal at C
35	Curvature of signal from start to Midpoint

### Supplemental oxygen

Oxygen insertion through the cannula may lower the CO_2_ concentration and lead to a significant error in FEV1 prediction. An analysis was made to investigate the effect of O_2_ on the model results, in thirteen patients who had waveform recordings with and without oxygen supplementation. Overall, oxygen supplementation did not cause a significant difference in predicted values (P=0.058).

## Discussion

In this study we have developed an automated method to evaluate the FEV1 of asthma and COPD patients, based on capnography waveforms. This method is based on quantitative characterization of waveform features and model generation with ANN. The model provides an output score that corresponds to the percent predicted FEV1 score measured with spirometry. Good correlation between the model results and actual %FEV1 was achieved. The creation of this model was challenging due to significant variance between waveforms detected during data collection; indeed, a large number of features was needed to detect such minimal changes, yet also be balanced with over fitting of the model. We found that the optimal number of features was 35, while higher numbers of features had resulted in over-fitting and less optimal model performance. Using this number of features allowed to identify minor changes in the waveform and provide high resolution in prediction. In addition, using demographic features, such as age, gender and height, provided additional information and improved prediction and model performance.

Several previous studies have shown the correlation between CO2 waveform and obstructive disease. You et al. analyzed the correlation between waveform features to spirometry indices. However, they performed manual waveform analysis on a small sample of 30 asthmatic patients and 10 healthy subjects [12]. Three other studies that used computerized methods, analyzed smaller sample sizes to differentiate between healthy, obstructive lung disease and chronic heart failure patients [13,16]. This is the first study that offers a method to predict the actual FEV1 value with a sample size adequate for validation.

This model has several practical medical uses. First, capnography assessment can be performed without reliance on patient cooperation. Second, the equipment required are a nasal canula and compatible monitoring, as opposed to spirometry, which requires more cumbersome equipment and an experienced technician. Moreover, since results, do not rely on patient cooperation, they will be repeatable and cannot be manipulated. Third, this prediction model allows for continuous monitoring, and thus closely following hospitalized patients with severe exacerbation, possibly allowing both shortened hospitalization and early recognition of deteriorating patients. Finally, in regard to the current COVID-19 pandemic, this method does not require a technician and can be done at tidal volume in an isolated room, thus, COVID-19 exposure is decreased substantially. Nevertheless, this model has several limitations that must be discussed: First, the model was not evaluated in pediatric patients, patients with severe disease who suffer from hypercapnic failure, hospitalized patients and mechanically ventilated patients. For model validation in these populations, careful assessment in future studies is needed. Second, the dataset included patients with known obstructive disease. Therefore, the effect of restrictive disease or a mixed obstructive-restrictive disease on the model is unknown. Consequently, its use is limited to patients with known obstructive disease, and it cannot be used for diagnosis or evaluation of patients with an unknown diagnosis. Third, we did not measure the diffusion capacity (DLCO) of participants and since the DLCO has an effect on the capnography waveform [25], it may also contribute to the models prediction ability. Future studies with this model should include DLCO to account for its impact. Finally, the influence of supplemental oxygen on model performance was not well studied. A paired analysis of 13 patients that required supplemental oxygen, with and without oxygen supplementation, did not show a difference in model performance (P=0.058). However, the confidence interval was close to statistical significance and in a larger sample size a clear difference may be seen. Therefore, the model cannot be used in patients with supplemental oxygen until the influence of oxygen is evaluated in future research.

## Conclusion

In this study we have developed a model to evaluate FEV1 in asthma and COPD patients. Using this model, as a point-of-care tool, we can evaluate the airway obstruction level without reliance on patient cooperation. Moreover, continuous FEV1 monitoring can identify disease fluctuations, response to treatment and guide therapy.

## Data Availability

All data relevant to the study are included in the article additional data requested will be available upon reasonable request.

## Abbreviations

COPD: Chronic obstructive pulmonary disease
FEV1: Forced expiratory volume in 1s
FVC: Forced vital capacity
%FEV1: Percent predicted FEV1
AHR: Airway hyperresponsiveness
MCT: Methacholine challenge test
EtCO_2_: End tidal CO_2_
ANN: Artificial neural network
MSE: Mean square error
